# Cross-cultural adaptation and validation of the Diabetic Foot Questionnaire (DiaFootQ) into Spanish Language

**DOI:** 10.1016/j.aprim.2026.103491

**Published:** 2026-04-08

**Authors:** María Ruiz-Muñoz, Francisco Javier Martínez-Barrios, Raúl Fernández-Torres, María José Vallejo-Herrera, Jonatan García-Campos, Manuel González-Sánchez

**Affiliations:** a Department of Nursing and Podiatry, Faculty of Health Sciences, University of Malaga, C/Arquitecto Francisco Peñalosa, 3, 29010 Malaga, Spain; b Department of Endocrinology and Nutrition, Regional Hospital of Malaga, Avda. Carlos Haya, 84, 29010, Spain; c Department of Behavioural Sciences and Health, Miguel Hernandez University, San Juan de Alicante, 03550 Alicante, Spain; d Department of Physiotherapy, Faculty of Health Sciences, University of Malaga, C/Arquitecto Francisco Peñalosa, 3, 29010 Malaga, Spain

**Keywords:** Diabetic foot, Validation, Questionnaire, Spanish, Pie diabético, Validación, Cuestionario, Español

## Abstract

**Objective:**

To perform a cross-cultural adaptation and validation of the Diabetic Foot Questionnaire (DiaFootQ) into Spanish (DiaFootQ-Sp).

**Design:**

Cross-cultural adaptation, validity and reliability study.

**Site:**

Diabetic Foot Unit (Regional University Hospital of Malaga, Spain), Podiatry Teaching Unit (University of Malaga, Spain) and private clinics across Spain.

**Participants:**

Adults over 18 years old with type 2 diabetes mellitus who were referred for diabetic foot examination or already enrolled with DFD.

**Methods:**

An observational study was conducted in two phases: (1) translation and cross-cultural adaptation of the DiaFootQ into Spanish and (2) psychometric validation of the DiaFootQ-Sp. Recommendations by ISPOR Task Force for Translation and Cultural Adaptation were followed. Reliability and validity were assessed using internal consistency (Cronbach's *α*), test–retest reliability (ICC), and construct validity through factor analysis. Criterion validity was evaluated by correlating DiaFootQ-Sp with the Foot Function Index (FFI), SF-12v2, and EuroQoL.

**Results:**

A total of *n* = 466 participants completed the study. The DiaFootQ-Sp showed strong internal consistency (Cronbach's *α* = 0.903) and high test–retest reliability (ICC = 0.854–0.997). Sensitivity analysis reported SEM = 2.195 and MDC90 = 5.121. Construct validity was confirmed (KMO = 0.677, Bartlett's test *p* < 0.001). Criterion validity showed significant correlations with the EuroQol-5D (*r* = 0.889) and SF-12 (*r* = 0.353). The mean completion time was 8.61 min.

**Conclusions:**

DiaFootQ was successfully adapted and validated for Spanish-speaking population, providing a useful and reliable tool to assess patients with DFD. The quality of care and prevention might be enhanced by implementing this instrument in clinical practice.

## Abstract (es)

**Objetivos:**

Realizar la adaptación transcultural y validación al español del Diabetic Foot Questionnaire (DiaFootQ), dando lugar al DiaFootQ-Sp.

**Diseño:**

Estudio de adaptación transcultural, validez y fiabilidad.

**Emplazamiento:**

Unidad de Pie Diabético (Hospital Regional Universitario de Málaga, España), Unidad Docente de Podología (Universidad de Málaga, España) y clínicas privadas en distintas regiones de España.

**Participantes:**

Personas adultas mayores de 18 años con diabetes mellitus tipo 2 remitidas para exploración del pie diabético o ya incluidas por enfermedad del pie diabético (DFD).

**Métodos:**

Se llevó a cabo un estudio observacional en dos fases: (1) traducción y adaptación transcultural del DiaFootQ al español y (2) validación psicométrica del DiaFootQ-Sp. Se siguieron las recomendaciones del ISPOR Task Force for Translation and Cultural Adaptation. La fiabilidad y validez se evaluaron mediante consistencia interna (α de Cronbach), fiabilidad test-retest (ICC) y validez de constructo mediante análisis factorial. La validez de criterio se analizó correlacionando el DiaFootQ-Sp con el Foot Function Index (FFI), el SF-12v2 y el EuroQoL.

**Resultados:**

Un total de 466 participantes completaron el estudio. El DiaFootQ-Sp mostró una elevada consistencia interna (α de Cronbach = 0.903) y una alta fiabilidad test-retest (ICC = 0.854–0.997). El análisis de sensibilidad indicó un SEM = 2.195 y un MDC90 = 5.121. La validez de constructo quedó confirmada (KMO = 0.677; prueba de Bartlett p < 0.001). La validez de criterio mostró correlaciones significativas con el EuroQol-5D (r = 0.889) y el SF-12 (r = 0.353). El tiempo medio de cumplimentación fue de 8.61 minutos.

**Conclusiones:**

El DiaFootQ fue adaptado y validado con éxito para la población hispanohablante, proporcionando una herramienta útil y fiable para la evaluación de pacientes con DFD. La implementación de este instrumento en la práctica clínica podría mejorar la calidad asistencial y las estrategias de prevención.

## Introduction

Diabetes mellitus (DM) is a metabolic disorder that affects 451 million individuals on a global scale.^1^ Diabetic foot disease (DFD) is defined as the infection, ulceration, or destruction of tissues of the foot within individuals bearing a diagnosis of DM. This affliction manifests in neuropathy and/or peripheral arterial disease affecting the lower extremities.^2^, ^3^ In Europe, its prevalence registers at 5.1%.^4^

One-third of the entire population with diabetes will suffer from a diabetic foot ulcer at some point in their lifetime; compounding this issue, over 50% of these afflicted individuals will suffer from infection, substantially increasing the likelihood of amputation.^3^, ^5^ The survival rates for amputees remain less than 50% within a mere 5 years, surpassing the mortality rates observed in most malignancies.^6^

The early identification of the risk of DFD as well as the assessment of patients already affected by the condition, is essential to strengthen prevention strategies in primary care units and to avoid further disease progression. To this end, there is a need for instruments capable of quantifying variables pertinent to the health status of these patients.^7^ These instruments can be categorized based on their reliance on data provided by the patients themselves, often termed patient-reported outcome measures (PROMs).^8^

Spanish is the third most spoken language in the world by native speakers.^9^, ^10^ Its presence in Europe and America makes it a language spoken by a heterogeneous population, in terms of cultural diversity and disparity of resources. The absence of PROMs designed specifically for DFD patients in Spanish represents a notable gap in healthcare provision.^11^ Only three questionnaires are available with this purpose in Spanish: two for the assessment of diabetic foot ulcers,^12^, ^13^ and one for the assessment of foot self-care.^14^

Lately, a multi-dimensional questionnaire that not only considers these features but also other important variables implied in DFD was developed in the English language: the Diabetic Foot Questionnaire (DiaFootQ). This impactful questionnaire seems crucial to be used in the Spanish-speaker population since DFD incidence and prevalence is growing especially in native speakers nations.^15^, ^16^ Dimensions such as lifestyle, function, footwear, and self-care are not addressed in any other available questionnaire. This makes the DiaFootQ a valuable tool in primary care settings, enabling physicians and nurses to assess patients with diabetes from a broader perspective and to refer them more accurately to specialized units.

The objective of this research is to perform a cross-cultural adaptation and validation of the DiaFootQ in Spanish language (DiaFootQ-Sp).

## Material and methods

### Study design

To carry out the cross-cultural adaptation and validation of the DiaFootQ into Spanish, an observational study divided into two main phases was developed: (1) translation and cross-cultural adaptation and (2) psychometric tests.

### Participants

Participants were enrolled from the Diabetic Foot Unit of the Department of Endocrinology and Nutrition, Regional University Hospital of Malaga, (Malaga, Spain), the Podiatry Teaching Unit at the University of Malaga (Malaga, Spain) or specialized private clinics in DFD across Spain. Participants provided informed consent with possibility of refusal.

The inclusion criteria for the participants were: adults (≥18 years) with no upper age limit; adequate reading skills and full comprehension of Spanish language; patients with DM who were diagnosed with DFD previously or were referred to be assessed in first instance. Exclusion criteria were history of major lower limb amputation (above the ankle joint); pregnancy; physical disabilities that implies use of wheelchair or in-bed patients and presence of cognitive impairment, dementia, or visual disabilities that could hinder their ability to respond.

### Translation and cross-cultural adaptation of the Diabetic Foot Questionnaire (DiaFootQ)

The 25-item DiaFootQ was developed to assess two key dimensions: (1) lifestyle and function and (2) footwear and foot self-care. This well-structured, valid, and reliable tool demonstrated strong internal consistency (*α* = 0.916), with item response values ranging from ICC = 0.862–0.998. External validity correlation levels varied between *r* = 0.386 and *r* = 0.888. The questionnaire was designed for efficient completion, allowing for the automatic calculation of both subcategory scores and the total score, ensuring practicality in clinical and research settings.^17^ The DiaFootQ scoring system assigns a value from 0 to 4 to each item, yielding a maximum total score of 100. Higher scores indicate a more desirable health status in patients with DFD, whereas lower scores reflect poorer outcomes.

To ensure terminological and conceptual equivalence in the DiaFootQ-Sp, the adaptation followed the guidelines of ISPOR Task Force for Translation and Cultural Adaptation^18^ and the World Health Organization (WHO).^19^ The process consisted of five steps: (1) translation of the DiaFootQ from English to Spanish by two independent, blinded native Spanish speakers; (2) comparison of both translations and agreement on a preliminary version; (3) back-translation from Spanish to English by two independent native English translators; (4) resolution of discrepancies by a committee of five experts, leading to a refined preliminary version; and (5) pilot testing of the preliminary DiaFootQ-Sp with 25 participants.

### Questionnaires used for criteria validity

#### Foot Function Index (FFI)

This self-reported questionnaire comprises 23 items categorized into three subdomains: pain, disability, and activity limitation, reflecting patient-reported values.^20^

#### Quality of life SF-12v2

The SF-12v2 is a 12-item questionnaire assessing health-related quality of life (HRQoL), generating two summary scores: the Physical Component Summary and the Mental Component Summary, both ranging from 0 to 100. It also includes eight domains: physical functioning, physical role, bodily pain, general health, vitality, social functioning, emotional role, and mental health. Summary scores and domain scores are computed using weighted algorithms, where each response contributes to the total score. Higher scores indicate better perceived HRQoL.^21^

#### EuroQoL Quality of Life Questionnaire (5-D and VAS)

The EuroQol-5D (EQ-5D) is a questionnaire designed to assess the quality of life, consisting of five domains: mobility, self-care, usual activities, pain/discomfort, and anxiety/depression. Each domain is rated on three severity levels: no problems, some/moderate problems, and severe problems. Additionally, it includes a visual analog scale (EQ-5D VAS) represented by a 10 cm vertical scale ranging from 0 (worst-perceived health) to 100 (best-perceived health).^22^

### Data collection

All participants completed the following questionnaires: DiaFootQ, FFI, SF-12v2, EuroQoL, and a sociodemographic survey. Based on previous studies indicating higher internal consistency and reliability with intervals of less than seven days, the DiaFootQ-Sp was administered twice, with a 3–5-day gap between measurements.^23^ To assess the construct validity of the DiaFootQ, FFI, SF-12v2, and EuroQoL questionnaires were utilized. Data collection took place between January 2023 and December 2024. Two blinded researchers, independent of the study, were responsible for both data collection and analysis. To estimate the sample size, ratio 10:1 (ten participants per item) was applied, with a minimum size of *n* = 250 participants required.^24^

### Data analysis

A frequency analysis was conducted to examine key characteristics of the sample, along with a descriptive analysis of sociodemographic variables and outcome measures (DiaFootQ, FFI, SF-12v2, and EuroQoL), calculating the mean and standard deviation. The Kolmogorov-Smirnov test was applied to assess the distribution and normality of the data (the significance level was set at *p* ≤ 0.05).

Cronbach's *α* coefficients were calculated to assess the internal consistency of the measures. Additionally, item response reliability was evaluated using the intraclass correlation coefficient (ICC – 2:1). Reliability values were classified as follows: poor (≤0.40), moderate (0.40–0.60), good (0.60–0.80), and excellent (≥ 0.80).^25^

The formula $\text{SEM}=s\sqrt{1-r}$ was used to calculate the standard error of measurement (SEM). For both measures (APFQ-Sp1 and APFQ-Sp2) the test score's standard deviation was “*s*”, and “*r*” was Pearson's correlation coefficient. Following the analysis described by Stratford,^26^ to measure the sensitivity of the tool, the minimal detectable change 90 (MDC90) was used. The formula used to calculate the MDC90 was as follows: MDC90 = SEM × √2 × 1.65. The floor or ceiling effect was present if more than 15% of the participants reached the lowest or highest score, respectively.

The validity of the construct was analysed using the maximum likelihood extraction (MLE) method. To preserve the original structure of the DiaFootQ, a two-factor forced model was applied. Additionally, the MLE was conducted while ensuring the requirement of at least 10 subjects per item was met.^27^ Kaiser–Meyer–Olkin test and Barlett's test of sphericity were calculated.

Criterion validity was calculated by analysing the degree of correlation between the DiaFootQ-Sp and the Spanish versions of the questionnaires: DiaFootQ, FFI, SF-12v2, and EuroQoL. Pearson's correlation coefficient was structured according to the following scale: *r* ≤ 0.49 (poor), 0.50 ≤ *r* ≤ 0.74 (moderate), *r* ≥ 0.75 (strong).^28^ To perform the statistical analysis of this study, the SPSS statistical treatment program (V.23.0) was used.

### Ethical considerations

This study involving human subjects was conducted in strict accordance with the ethical principles outlined in The Code of Ethics of the World Medical Association (Declaration of Helsinki) for experiments involving humans. The research protocol was reviewed and approved by Ethics Committee of the University of Malaga under protocol number UVIC-CCC 81/2019.

Data handling complied with Organic Law 3/2018 on the Protection of Personal Data and the guarantee of digital rights.

## Results

The translated version of the DiaFootQ into Spanish (DiaFootQ-Sp) is available in the Supplementary File. A total of *n* = 466 people participated in this study. Table 1 shows the descriptive characteristics of the sample by frequency of occurrence. There is a balance in the distribution of participants by biological sex (45.3 vs. 54.7). The level of education is balanced between the three main levels, ranging from 26.0% to 33.7%. On the other hand, almost half of the participants were receiving oral treatment, while more than a quarter of the sample (26.4%) were receiving combined treatment (oral and insulin). Only 3.4% of participants received no treatment.

**Table 1:** Qualitative descriptive characteristics of the sample.

	Frequency	Percentage	Cumulative percentage
*Sex*			
Male	211	45.3	45.3
Female	255	54.7	100

*Education level*			
Primary education	157	33.7	33.7
Secondary education	121	26.0	59.7
Higher education	138	29.6	89.3
Others	41	8.9	98.2
None	9	1.8	100

*Pharmacological treatment*			
Oral	77	16.6	16.6
Insulin	209	44.8	61.4
Both	123	26.4	87.8
Others	41	8.8	96.6
None	16	3.4	100

N	466		

Table 2 shows the descriptive characteristics of the sample, with the mean, minimum, and maximum values recorded and the standard deviation. The mean age recorded in the group is 57.87 years, while the mean number of months since the sample was diagnosed with DM is 136.24 months, which is more than 11 years on average.

**Table 2:** Quantitative descriptive characteristics of the sample.

	Min	Max	Mean	Standard deviation
*Age (years)*	38	86	57.87	12.86
*Duration of diabetes mellitus (months)*	11	255	136.24	69.56
*DiaFootQ-Sp*	31	100	66.02	12.20
*EsDQoL*	4	160	95.11	28.08
*DFS SF*	0	114	58.05	19.01

*FFI*				
Index	65	33.20	18.12	18.53
Pain	41	14.29	11.46	11.61
Disability	47	15.64	12.01	12.26
Limit activity	17	5.34	3.88	4.03

*SF-12*				
Physical function	67.16	47.49	13.72	14.83
Role physical	62.91	34.46	12.06	12.91
Bodily pain	63.90	33.45	14.71	12.53
General health	64.61	42.66	12.68	18.81
Vitality	67.88	49.32	12.26	17.37
Social functioning	65.70	40.99	18.99	12.99
Role emotional	68.81	34.78	17.31	17.03
Mental health	65.73	42.40	13.07	12.18
Physical component state	64.84	39.04	16.77	14.11
Mental component state	75.48	51.08	11.87	12.23

*EuroQol VAS*	28.00	97.00	76.40	16.57
*EuroQol 5D*	0.280	1.000	0.77	0.17
*n*	466			

When analysing the ceiling and floor effects, it was observed that *n* = 22 (4.7%) and *n* = 17 (3.6%) reached the maximum and minimum score respectively, so based on the observed results, the ceiling/floor effect is not considered relevant for the DiaFootQ-Sp.

The mean time to complete the questionnaire was 8.61 min. The internal consistency of the DiaFootQ-Sp was Cronbach's alpha = 0.903, while the interclass correlation indices of the item responses ranged from 0.854 (item 6) to 0.997 (item 24). The calculation of the sensitivity measures showed values of SEM = 2.195 and MDC90 = 5.121.

The construct validity, whose extraction method was maximum likelihood, showed values of 0.677 in the Kaiser–Meyer–Olkin test, with a chi-square value of 3253.984 with 300 degrees of freedom and a significant value in Barlett's test of sphericity (*p* < 0.001).

**Figure 1:**
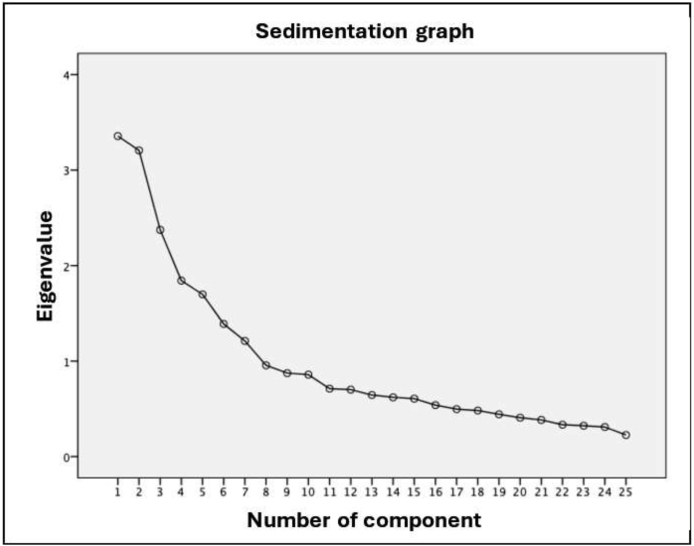
Sedimentation graph of the DiaFootQ-Sp.

The DiaFootQ-Sp represents a two-factor solution, as only two factors met one of the extraction requirements (13.426% and 12.828%, for factors 1 and 2 respectively). Fig. 1 shows the sedimentation plot, while Table 3 presents the eigenvalue and the variance explained for each of the items that make up the questionnaire (in the variance explained, only those items that exceed 10% are presented).

**Table 3:** Eigenvalues and variance explained by each of the items of the DiaFootQ-Sp.

Component	Initial eigenvalues			Extraction sums of squared loads		
	Total	% of variance	% cumulative	Total	% of variance	% cumulative
1	3.357	13.426	13.426	3.357	13.426	13.426
2	3.207	12.828	26.254	3.207	12.828	26.254
3	2.375	9.499	35.753			
4	1.843	7.371	43.125			
5	1.699	6.795	49.919			
6	1.389	5.557	55.477			
7	1.211	4.845	60.322			
8	.956	3.822	64.144			
9	.875	3.498	67.642			
10	.858	3.434	71.076			
11	.711	2.844	73.919			
12	.701	2.804	76.723			
13	.645	2.578	79.301			
14	.621	2.483	81.785			
15	.607	2.429	84.214			
16	.539	2.157	86.370			
17	.497	1.988	88.358			
18	.482	1.930	90.288			
19	.443	1.771	92.059			
20	.407	1.630	93.689			
21	.384	1.535	95.224			
22	.334	1.338	96.562			
23	.324	1.295	97.857			
24	.310	1.239	99.095			
25	.226	.905	100.000			

For the analysis of criterion validity, both the total score of the DiaFootQ-Sp and the two extracted factors were used. The significant correlation values of the DiaFootQ-Sp ranged from *r* = 0.889 (EuroQol-5D) to *r* = 0.353 (SF-12 – General Health) (Table 4).

**Table 4:** Correlation between the DiaFootQ-Sp and the used tools for criteria validity analysis.

	DiaFootQ_Sp Total	DiaFootQ_Sp Factor 1	DiaFootQ_Sp Factor 2
*SF-12*			
Physical function	0.556*	0.166*	0.417*
Role physical	0.528*	0.152*	0.394*
Bodily pain	0.507*	0.207*	0.436*
General health	0.353*	0.168*	0.241*
Vitality	0.594*	0.084	0.590*
Social functioning	0.477*	0.149*	0.373*
Role emotional	0.564*	0.075	0.453*
Mental health	0.521*	0.058	0.414*
Physical component state	0.607*	0.120*	0.453*
Mental component state	0.723*	0.183*	0.586*

*EuroQol_VAS*	0.873*	0.227*	0.689*
*EuroQol_5D*	0.889*	0.228*	0.703*
*EsDQoL*	0.786*	0.124*	0.637*
*DFS-SF*	−0.820*	−0.079	−0.672*

*FFI*			
Index	−0.840*	−0.282*	−0.639*
Pain	−0.760*	−0.075	−0.671*
Disability	−0.795*	−0.123*	−0.696*
Limit_Activity	−0.852*	−0.239*	−0.668*

* *p* < 0.05.

## Discussion

The study aimed to carry out a cross-cultural validation of the DiaFootQ questionnaire in Spanish. The translation and validation followed the recommendations of the literature to ensure conceptual equivalence between the original English version and this new Spanish version.

The DiaFootQ-Sp showed excellent statistical properties, particularly in terms of construct and criterion validity. These data suggest that the instrument is valid and reliable for use in Spanish-speaking populations, as it ensures that the specific cultural and linguistic characteristics of Spanish-speaking patients have been adequately taken into consideration, improving the accuracy and relevance of the data collected.

To date, this is the first cross-cultural validation study of the DiaFootQ in another language. The DiaFootQ-Sp joins other PROMs already validated in Spanish that are closely related to DFD, such as the Diabetic Foot Ulcer Scale – Short Form (DFUS-SF),^13^ the Questionnaire for Diabetes-Related Foot Disease (QDFD),^29^ the Foot Health Status Questionnaire (FHSQ),^30^ the Cardiff Wound Impact Schedule Questionnaire (CWISQ)^12^ and the Multicultural Quality of Life Index (MQLI).^31^

The DFUS-SF provides a detailed measure of symptoms and quality of life related to DFD. The Spanish validation study used a sample of *n* = 141 with a Cronbach's alpha = 0.720–0.948, an ICC = 0.77–0.92 and excellent criterion validity.^13^

The QDFD is a PROM that focuses on patient reports of symptoms caused by peripheral arterial disease and diabetic neuropathy. The Spanish validation study used a sample of *n* = 92. Criterion validity showed a kappa coefficient of 0.823.^29^

The FHSQ provides a broader assessment of foot health which was not developed for patients with DM. In its transcultural validation to Spanish, only content validity was analysed.^30^

The CWISQ and the MQLI also provide valuable information regarding the assessment of the impact of diabetic foot ulcers. The CWISQ validation study shows a sample of *n* = 141, excellent content validity, Cronbach's alpha = 0.715–0.797, ICC = 0.63 and excellent criterion validity. The MQLI does not provide specific information on DFD.^31^

Most of these above-mentioned questionnaires, presented insufficient statistical robustness in their validation studies compared to DiaFootQ-Sp; however, the Diabetic Foot Self-Care Questionnaire (DFSQ-UMA)^14^ presented excellent psychometric properties and was developed in Spanish, although it is exclusively focused in self-care outcomes. On the contrary, DiaFootQ-Sp includes items that concerns self-care attitudes apart from other regarding physical abilities, footwear, pain, sociocultural impact and knowledge, making this questionnaire a more complete option.

In Spain, it is estimated that around 1,500,000 people suffer from some manifestation of diabetic foot disease, including diabetic neuropathy and peripheral arterial disease.^32^ In Latin America, the prevalence of diabetic foot disease varies widely, ranging from 3% to 36.5%.^33^ This variability reflects differences in access to care and diabetes prevention and management strategies. The absence of an adequate instrument for assessing DFD in Spanish may be related to the lack of established prevention protocols within health systems, unfavourable working environments and detrimental administrative decisions.

The availability of the DiaFootQ in Spanish as a standardised tool allows for a more accurate assessment of patients, improving the detection of risks, complications and needs leading to the implementation of appropriate interventions. The early detection of risk patients based on low scores of this PROM should trigger referring to endocrinology, nursing or podiatry units in cases where secondary prevention level is required. Its use in daily clinical practice, especially in primary care units can contribute to reducing the incidence of ulcers and amputations, optimising patients’ quality of life and reducing the costs associated with diabetic foot care.

The adaptation process of the DiaFootQ included critical stages such as translation and back-translation, as well as validation by a panel of experts and pilot testing on a representative sample. Statistical analyses confirmed the internal consistency and reliability of the questionnaire, in line with the standards set by previous cross-cultural adaptation studies.

It should be noted that the use of a cross-culturally adapted questionnaire, such as the DiaFootQ, can facilitate the comparison of data between different studies and populations, thus promoting a better global understanding of foot health in people with DM.

Despite these advances, the study has some limitations. Results reports were not disaggregated by sex, missing out possible differences in the descriptive outcomes. Since part of the participants were recruited from specialized units, it might have affected harder-to-reach patients, introducing a potential selection bias. Furthermore, recruitment was conducted in a single region of the country, thus limiting geographic diversity and generalizability.

Gender identity was not reported since it was not considered to influence psychometric properties, however, it is advisable to be assessed in future research, likewise demographic variables regarding glycemia, HbA1c, or diabetes-related complications. Due to the recent creation and validation of the DiaFootQ in its original language (English), it has not yet been used in RCTs or longitudinal studies, and therefore its clinical value remains to be tested in future research.

In conclusion, the cross-cultural adaptation of the DiaFootQ questionnaire into Spanish and its validation was successful, providing a useful and reliable tool to assess foot health in patients with DFD in Spanish-speaking contexts. The integration of this questionnaire into primary care assessment and research may significantly improve the quality of care and outcomes for these patients.

## CRediT authorship contribution statement

Conceptualization, M.R., M.V.H., M.G.; methodology F.J.M.B., M.G.; software, R.F., F.J.M.B.; validation, M.R., R.F.; formal analysis, J.G.C.; investigation, R.F.; resources, M.R., M.G.; data curation, M.R., J.G.C.; writing – original draft preparation, R.F., M.V.H., M.G., M.R. FJ.M.B.; writing – review and editing, M.V.H., J.G.C., A.G.M.; visualization, R.F., J.G.C.; supervision; project administration, C.F., F.J.M.B., M.R.

All authors have read and agreed to the published version of the manuscript.

## Patient consent

All participants provided informed consent.

## Declaration of generative AI and AI-assisted technologies in the writing process

No generative AI nor AI-assisted technologies were used in the writing process of this manuscript.

## Funding

This research did not receive any specific grant from funding agencies in the 248 public, commercial or not-for-profit sectors.

## Conflicts of interest

All authors declare that they have no conflict of interests.

## Data availability

The data used in this study are available from the corresponding author upon reasonable request. Due to privacy/ethical restrictions, the data cannot be made publicly available.
