# Supplementary material for: Cross-cultural adaptation and validation of the Diabetic Foot Questionnaire (DiaFootQ) into Spanish Language
Source: Aten Primaria. 2026 Apr 8;58(6):103491. doi: 10.1016/j.aprim.2026.103491 (PMC13091101; doi:10.1016/j.aprim.2026.103491)
Supplement: Supplementary file 1 [file mmc1.doc]

# DIAFOOTQ-Sp

Estas preguntas se refieren al impacto que su enfermedad de pie diabético puede tener en su vida diaria y su bienestar. Responda a cada pregunta y marque solo un número en cada línea. Si no está seguro de alguna respuesta, marque la más precisa o pregunte al investigador.

# 0 1 2 3 4

| Nunca | Muy poco | A veces | A menudo | Siempre |
| --- | --- | --- | --- | --- |
|  |  |  |  |  |
|  |  |  |  |  |
|  |  |  |  |  |
|  |  |  |  |  |
|  |  |  |  |  |

¿Con qué frecuencia...

1. **Revisa usted sus pies?**
2. **Revisa usted su calzado (interior y exterior)?**
3. **Seca entre los dedos de los pies después de mojarlos?**
4. **Usa crema hidratante en sus pies?**
5. **Visita a su podólogo?**

¿Cuánta dificultad tiene para ...

| Mucha | Bastante | Normal | Poca | Ninguna |
| --- | --- | --- | --- | --- |
|  |  |  |  |  |
|  |  |  |  |  |
|  |  |  |  |  |
|  |  |  |  |  |

1. **Lavarse y secarse los pies?**
2. **Cortarse las uñas de los pies?**
3. **Encontrar un zapato adecuado (ancho, contrafuerte, materiales naturales, con sujección, etc.) para sus pies?**
4. **Realizar ejercicio físico como senderismo, ciclismo, natación, gimnasio, etc.**

Debido a sus problemas en los pies...

| Mucha | Bastante | Normal | Apenas | Ninguna |
| --- | --- | --- | --- | --- |
|  |  |  |  |  |
|  |  |  |  |  |
|  |  |  |  |  |
|  |  |  |  |  |

1. **¿Qué dificultad tiene para caminar 400 m?**
2. **¿Qué dificultad tiene para subir o bajar dos pisos de escaleras?**
3. **¿Necesita ayuda externa para su aseo e higiene personal? (una persona, bastón, silla etc.)**
4. **¿Cuánta limitación tiene para realizar sus actividades de la vida diaria (cocinar, levantarse por la mañana, tareas domésticas...)**

| Mucho | Bastante | Normal | Apenas | Nada |
| --- | --- | --- | --- | --- |
|  |  |  |  |  |
|  |  |  |  |  |
|  |  |  |  |  |
|  |  |  |  |  |
|  |  |  |  |  |
|  |  |  |  |  |

¿Cuánto le afectan tus problemas en los pies

en cuanto a

1. **¿Su vida laboral (ahora o antes)?**
2. **¿Su nivel económico?**
3. **¿Sus relaciones sociales?**
4. **¿Su estado de ánimo?**
5. **¿Su sueño?**
6. **¿Su calidad de vida?**

¿Considera que...

| Nada | Muy poco | Normal | Bastante | Mucho |
| --- | --- | --- | --- | --- |
|  |  |  |  |  |
|  |  |  |  |  |
|  |  |  |  |  |
|  |  |  |  |  |

1. **La dieta es importante para la salud de sus pies?**
2. **Practicar actividad física es bueno para sus problemas en los pies?**
3. **El calzado es importante para la salud de sus pies?**
4. **El uso de elementos ortopédicos (plantillas, elevadores de talón, protectores de silicona, zapatos a medida, etc.) mejora la salud de sus pies?**
5. **¿Cuánto se preocuparías si apareciera alguna herida o úlcera en su pie?**

| Mucho | Bastante | Normal | Apenas | Nada |
| --- | --- | --- | --- | --- |
|  |  |  |  |  |
|  |  |  |  |  |

1. **¿Cuánto dolor tiene en los pies a consecuencia de la diabetes?**

**PUNTUACIÓN TOTAL: / 100**
